# Continued care and provision of glasses are necessary to improve visual and academic outcomes in children: Experience from a cluster-randomized controlled trial of school-based vision screening

**DOI:** 10.17269/s41997-024-00884-8

**Published:** 2024-04-30

**Authors:** Mayu Nishimura, Agnes Wong, Daphne Maurer

**Affiliations:** 1 Department of Psychology, Neuroscience & Behaviour, McMaster University, Hamilton, ON Canada; 2 Department of Ophthalmology and Vision Sciences, The Hospital for Sick Children, Toronto, ON Canada; 3 Department of Ophthalmology and Vision Sciences, University of Toronto, Toronto, ON Canada; 4 Dalla Lana School of Public Health, University of Toronto, Toronto, ON Canada

**Keywords:** Amblyopia (developmental), Refractive errors, Vision screening, Strabismus, Amblyopie (développementale), troubles de la réfraction oculaire, dépistage visuel, strabisme

## Abstract

**Objective:**

To assess the effectiveness of a kindergarten vision screening program by randomly assigning schools to receive or not receive vision screening, then following up 1.5 years later.

**Methods:**

Fifty high-needs elementary schools were randomly assigned to participate or not in a vision screening program for children in senior kindergarten (SK; age 5‒6 years). When the children were in Grade 2 (age 6‒7 years), vision screening was conducted at all 50 schools.

**Results:**

Contrary to expectations, screened and non-screened schools did not differ in the prevalence of suspected amblyopia in Grade 2 (8.6% vs. 7.5%, *p* = 0.10), nor prevalence of other visual problems such as astigmatism (45.1% vs. 47.1%, *p* = 0.51). There was also no difference between screened and non-screened schools in academic outcomes such as the proportion of children below grade level in reading (33% vs. 29%) or math (44% vs. 38%) (*p* = 0.86). However, more children were wearing glasses in screened than in non-screened schools (10.2% vs. 7.8%, *p* = 0.05), and more children reported their glasses as missing or broken (8.3% vs. 4.7%, *p* = 0.01), suggesting that SK screening had identified successfully those in need of glasses. Examination of individual results revealed that 72% of children diagnosed and treated for amblyopia in SK no longer had amblyopia in Grade 2.

**Conclusion:**

The prevalence of amblyopia and other visual problems was not reduced in Grade 2 by our SK vision screening program, perhaps because of poor treatment compliance and high attrition. The results suggest that a single screening intervention is insufficient to reduce visual problems among young children. However, the data from individuals with amblyopia suggest that continuing vision care and access to glasses benefits children, especially children from lower socioeconomic class.

## Résumé

**Objectif:**

Évaluer l’efficacité d’un programme de dépistage visuel à l’école maternelle (EM) en assignant aléatoirement des écoles à participer ou non à un tel programme, puis en faisant un suivi un an et demi après.

**Méthodes:**

Cinquante écoles primaires pour étudiants et étudiantes ayant des besoins importants ont été assignées aléatoirement à participer ou non à un programme de dépistage visuel auprès des enfants fréquentant la maternelle (EM; 5‒6 ans). Lorsque ces enfants étaient en 2^e^ année (6‒7 ans), un dépistage visuel a été effectué dans les 50 écoles.

**Résultats:**

Contre toute attente, il n’y a pas eu de différence entre les écoles ayant participé ou non au dépistage dans la prévalence de l’amblyopie présumée en 2^e^ année (8,6 % contre 7,5 %, *p* = 0,10), ni dans la prévalence d’autres problèmes de vision comme l’astigmatisme (45,1 % contre 47,1 %, *p* = 0,51). Il n’y a pas eu non plus de différence dans les résultats scolaires des deux groupes d’écoles, comme la proportion d’enfants dont le niveau en lecture (33 % contre 29 %) ou en mathématiques (44 % contre 38 %), *p* = 0,86, ne correspondait pas à leur année d’étude. Cependant, le nombre d’enfants portant des lunettes était plus élevé dans les écoles ayant participé au dépistage que dans les autres écoles (10,2 % contre 7,8 %, *p* = 0,05), ainsi que le nombre d’enfants disant avoir perdu ou brisé leurs lunettes (8,3 % contre 4,7 %, *p* = 0,01), ce qui indique que le dépistage en maternelle a identifié avec succès les enfants ayant besoin de lunettes. L’examen des résultats individuels a révélé que 72 % des enfants diagnostiqués et traités pour l’amblyopie en maternelle ne présentaient plus d’amblyopie en 2^e ^année.

**Conclusion:**

Notre programme de dépistage visuel à l’école maternelle n’a pas réduit la prévalence de l’amblyopie et d’autres problèmes de vision en 2^e^ année, peut-être en raison du manque d’assiduité au traitement et d’une attrition importante. Les résultats indiquent qu’une seule intervention de dépistage ne suffit pas à réduire les problèmes de vision chez les jeunes enfants. Cependant, les données individuelles des sujets présentant une amblyopie indiquent qu’il est avantageux pour les enfants, et surtout ceux de la classe socioéconomique inférieure, de continuer de recevoir des soins de la vue et d’avoir accès à des lunettes.

## Introduction

Visual problems of concern among young children are amblyopia and refractive errors. Amblyopia is reduced vision (usually in one eye) in an otherwise normal eye that results most commonly from strabismus (eye misalignment) or anisometropia (unequal refractive errors between eyes) in early childhood that impaired the brain’s visual processing (Hutchinson et al., 2022; Quinlan & Lukasiewicz, 2017). Estimates of prevalence range from 1.8% to 5.4% in children under 7 years of age (Drover et al., 2008), although the rate is higher at 7.7% in children from low-income families (Pascual et al., 2014). Refractive errors cause blurred vision and are also risk factors for developing amblyopia (Pascual et al., 2014). Refractive errors include anisometropia, hyperopia (far-sightedness), myopia (near-sightedness), and astigmatism (blurred vision along one axis). Estimates of prevalence in children under 7 range from 0.6% to 8.9%, depending on the type of refractive error and ethnicity (Drover et al., 2008; Giordano et al., 2009).

Amblyopia, if left untreated, can result in reading impairment (Kelly et al., 2015; Shankar et al., 2007; VIP-HIP Study Group, 2016), and in worst cases, blindness (Wong, 2012). Treatment for amblyopia is more effective if started before age 7 (Chen & Cotter, 2016; Holmes et al., 2011), making it critical to identify and treat amblyopia (and its risk factors) before age 7. Historically, the provincial Ontario Health Insurance Plan (OHIP) has paid for children’s eye exams by eye care professionals (i.e., optometrists and ophthalmologists). However, 35% of children born in 2010 (before public health mandated vision screening) never had an eye exam from birth to age 7, a proportion that rises to ~ 50% in children from low-income families (Asare et al., 2022). These findings suggest that eye care is under-utilized, and visual problems are undetected. Once under the care of an optometrist/ophthalmologist, children can receive treatments for amblyopia, such as patching the weaker eye or wearing glasses to send clear visual signals to the brain, and these treatments can reverse amblyopia (Chen & Cotter, 2016; Holmes et al., 2011). Anisometropia and high refractive are also risk factors for amblyopia, so treatment with glasses not only helps children see more clearly (which can aid reading; Roch-Levecq et al., 2008) but also *prevents* amblyopia from developing (Koo et al., 2017).

Although universal screening appears to be a necessary first step in getting children into eye care, systematic reviews of the literature have not found conclusive evidence that vision screening is effective (Bennett & Maloney, 2017; Jonas et al., 2017; Public Health Ontario, 2016), mainly because the findings are correlational and observational or lack random assignment to a “no screening” control group. Despite this evidentiary gap, a precautionary approach has led organizations, including the Canadian Association of Optometrists, the Canadian Ophthalmological Society (Delpero et al., 2019), the World Health Organization (WHO Press, 2007), the American Association for Pediatric Ophthalmology and Strabismus (AAPOS, 2022), the US Preventive Services Task Force (US Preventive Services Task Force, 2017), and the Canadian Paediatric Society (Amit et al., 2009), to recommend vision screening for children aged 3‒5 years. Therefore, in 2018, Ontario modified its public health standards to mandate vision screening for children in senior kindergarten (i.e., children turning 5 that calendar year). Such a screening program *identifies* children who are at risk for visual problems who should be referred to an optometrist/ophthalmologist. From a public health perspective, the assumption is that *parents* will receive the referral letter and then follow up with eye care professionals to ensure their children receive *treatment*. It is unclear to what extent parents will follow up, and a further complication with the Ontario model is that OHIP covers the cost of eye exams, but not the cost of glasses (the most common treatment).

**Fig. 1:**
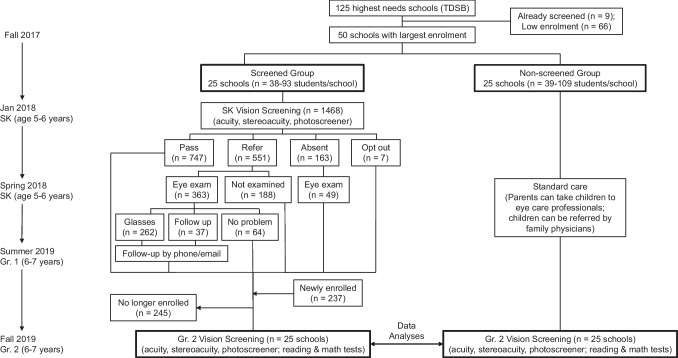
Schematic diagram of sequence of events for screened and non-screened schools

Given such difficulties, skepticism remains among relevant individuals and professionals (parents, clinicians, educators, and health policy makers) about whether school-based screening actually *reduces* the prevalence of amblyopia and uncorrected refractive errors, which can only be estimated at the population level. As a first step, we used a cluster-randomized controlled design (Fig. 1) to determine whether the effectiveness of a comprehensive screening program can be shown at the *school* level rather than focusing on the efficacy of individual treatment. We offered screening and comprehensive eye exams (including any needed glasses) to senior kindergarten children in 25 schools, and then later, when children were in Grade 2, compared estimates of amblyopia in those screened schools relative to 25 comparison schools. Vision screening occurred before the implementation of the Ontario public health vision screening mandate that began in 2018. We chose children in senior kindergarten to align with this mandate, and targeted high-needs schools (identified by the school board) to maximize the benefits to under-privileged children.

## Methods

### Design and participants

The research protocol was approved by the research ethics boards of the Toronto District School Board (TDSB), The Hospital for Sick Children, and the Hamilton Integrated Research Ethics Board.

#### Design

We used a cluster-randomized trial in which the prevalence of visual disorders was compared between screened vs. non-screened schools given and not given the screening intervention, 1.5 years later. The screening intervention comprised screening, referral for children who did not pass screening, and offer of eye exams for referred children.

#### Selection of clusters

We chose “schools” as our clusters. Because our study site was Toronto, Ontario, we used a measure developed by the Toronto District School Board called the Learning Opportunities Index (LOI), a needs-based criterion to rank schools based on external challenges affecting student success (e.g., family income, adults with low education, non two-parent families, and % of families receiving social assistance; https://www.tdsb.on.ca/research/research/learning-opportunities-index). The top 125 schools with highest needs receive the Model Schools for Inner Cities (MSIC) program, which offers supports, such as the Gift of Sight & Sound of the Toronto Foundation for Student Success, with whom we collaborated.

#### Sample size calculations

Based on previous research (e.g., Donahue et al., 2013; Nishimura et al., 2020), we estimated the prevalence of amblyopia to be 6% in low-income neighbourhoods. To demonstrate a significant difference between screened and non-screened schools (intraclass correlation coefficient of 0.01) with a *predicted* prevalence of 3% (i.e., a successful screening program should reduce the prevalence of amblyopia by half), we needed 25 schools (clusters) per group (screened vs. non-screened), and 40 children per school.

#### Randomization process and selection of clusters

We chose the 50 MSIC schools with the highest senior kindergarten (SK) enrollment (38‒109 students per school), excluding nine schools that had received vision screening from our pilot study or ad hoc volunteer screening (see Fig. 1). A biostatistician who was not part of data collection randomly assigned (without any restrictions) half the schools to the “screened” group and half to the “non-screened” group. There was no systematic difference between screened and non-screened schools in LOI ranking (*t*(48) =  − 0.13, *p* = 0.90), nor LOI score (*t*(48) = 0.13, *p* = 0.90), suggesting that the schools in the “screened” and “non-screened” groups did not differ meaningfully at baseline.

#### Selection of individuals in schools

Parents received information letters 1‒2 weeks prior to screening and could opt out. All children whose parents did not opt out were eligible for screening and verbal assent was obtained from each child by asking if they were ready to play a “game to check how well you can see”.

### Intervention

#### Screening

In “screened” schools, we screened all SK children (whose parents did not opt out) using three tools validated in a previous study (Nishimura et al., 2019): HOTV crowded acuity cards, Randot Preschool Stereoacuity test, and Plusoptix photoscreener. Screeners underwent training offered by the authors. Children who met the referral criteria (Table 1) selected from AAPOS 2013 guidelines (Donahue et al., 2013) and age-appropriate norms (Birch et al., 2008), and children who could not complete a test (The Vision in Preschoolers Study Group, 2007), were referred for a comprehensive eye exam.

**Table 1:** Referral criteria for vision screening of children in senior kindergarten

Screening test		Referral criterion
HOTV Crowded Acuity Cards		Worse than 0.1 logMAR acuity in either eye
Plusoptix photoscreener	Hyperopia	> 2.0 D
	Myopia	< − 1.5 D
	Astigmatism	> 1.5 D
	Anisometropia IOD	> 1.5 D
Randot Preschool Stereoacuity		Worse than 60 arcsec

#### Eye exams

Appointments were made for each student who failed screening to receive a comprehensive eye exam by a licensed optometrist at the school, with a parent or guardian present (an additional consent form was signed by parents/guardians prior to the eye exam). When space was available, we also offered appointments to children who were absent on the day of screening (their data are not included in the analyses because they missed screening). Providing the eye exam is not a part of the current Ontario vision screening mandate; however, our rationale was to facilitate the follow-up by parents needed to ensure children *identified* through screening were *treated* for their visual problems. We encouraged parents who could not attend the appointment to book an appointment at the optometrist’s office. We recruited local optometrists with offices close to the school while giving priority to those participating in the Eye See…Eye Learn® program of the Ontario Association of Optometrists. We used AAPOS (Donahue et al., 2013) guidelines and the clinical judgement of the authors to classify visual problems (Table 2).

**Table 2:** Classification of visual problems for children in senior kindergarten

Disorder	Definition	
Amblyopia	** ≥ **2-line difference in best corrected acuity and worse than 20/40 in one eye	
Binocular vision		
1. Strabismus	Tropias > 10 D	
2. Reduced stereoacuity	> 100 arcsec	
Refractive errors	> 48 months	
1. Hyperopia (Sph)	> 3.5 D	
2. Astigmatism (Cyl)	> 1.5 D	
3. Anisometropia (SE)	> 1.5 D IOD	
4. Myopia (Sph)	< − 1.5 D	
Other problems	Nystagmus, vitreous abnormalities, optic nerve abnormalities	

#### Treatment with glasses

Opticians and needed glasses were provided through the Gift of Sight & Sound program, dispensed 2‒3 months after screening.

#### Follow-up

For children who received glasses or needed follow-up, we attempted to contact parents a year later to remind them of follow-up appointments and to offer assistance (e.g., replacement of lost glasses, rescheduling of appointments). However, we were mostly unsuccessful in contacting parents/guardians by phone (straight to voicemail or no longer in service) and we received only 7 responses out of 202 attempts by email.

### Variables and measurements

Our primary visual outcome was the prevalence of amblyopia, calculated at the *school* level (i.e., what % of Grade 2 children enrolled at the school has amblyopia?). Amblyopia was measured using an HOTV eye chart (see Table 3 for classification of visual disorders). A new team of screeners, who were masked to the allocation of schools to “screened” or “non-screened” groups, were hired and trained by the authors.

**Table 3:** Classification of visual disorders for children in Grade 2

Amblyopia (primary clinical outcome)
Bilateral amblyopia: acuity worse than 20/40 in better seeing eye
Unilateral amblyopia: 2-or-greater line difference in acuity with worse than 20/25 in at least 1 eye
Other visual problems (secondary clinical outcomes)
Reduced stereo: > 40 arcsec
Reduced acuity: worse than 20/25 in either eye
Refractive errors
Hyperopia ≥ 1.25 D
Myopia ≤ − 1.25 D
Astigmatism ≥ 0.75 D
Anisometropia Spherical hyperopic ≥ 1.00 D Spherical myopic ≥ 2.00 D Cylindrical ≥ 1.50 D

Our secondary outcome was % of children reading below grade level. The MSIC program collects reading and math scores annually. We requested Grade 2 scores for the 50 schools in our sample, which were collected in Fall 2019, the same period as our Grade 2 vision screening. Because the data were given to us aggregated at the school level (clusters), these scores included children who were not screened.

Visual problems other than amblyopia were measured using a Randot Stereoacuity test and Spot Vision Screener.

### Analysis

We conducted *t*-tests to compare the 25 screened and 25 non-screened schools on the prevalence of amblyopia and the prevalence of visual disorders other than amblyopia (we had planned on examining cluster effects, such as intraclass correlation, only if the *t*-tests suggested a difference between groups). We used a one-way ANOVA to compare screened vs. non-screened schools on reading and math scores, using the data on proportion of students who were below one or two standard deviations.

## Results

### SK vision screening

**Fig. 2:**
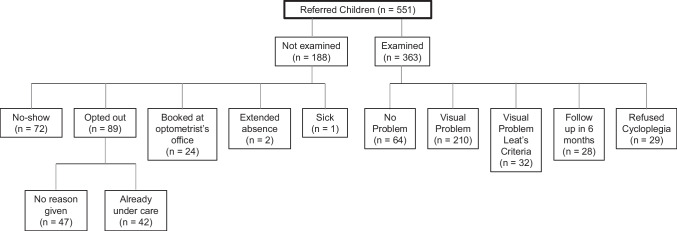
Description of SK children who failed visual screening

A total of 1468 children were eligible for screening, of whom 747 (50.9%) passed, 551 (37.5%) failed, 163 (11.1%) were absent, and 7 (0.05%) opted out of screening. We offered optometry exams to all 551 children who failed screening and the results are summarized in Fig. 2. We also examined an additional 49 children absent for screening because appointments were available, of whom 15 children were diagnosed with a visual problem; however, because they missed screening, they are not included in the counts of visual problems shown in Table 4. Amblyopia was diagnosed in 149 children (11.5% of screened) and 147 (11.3%) of them were prescribed glasses. An additional 115 (8.9%) children were prescribed glasses for refractive errors based on the prescribing guidelines by Leat (2011).

**Table 4:** Summary of the visual problems identified through comprehensive eye exams in senior kindergarten

Visual problems (*n* = 242)	Counts (% screened)
Amblyopia	149 (11.5%)
Bilateral	115 (8.9%)
Unilateral	34 (2.6%)
Risk factors*	
Reduced stereo	15 (1.2%)
Strabismus	8 (0.6%)
Astigmatism	42 (3.2%)
Hyperopia	9 (0.7%)
Myopia	6 (0.5%)
Anisometropia	6 (0.5%)
Glasses prescribed	147 (11.3%)
Glasses prescribed (Leat, 2011)	115 (8.9%)

*Excluding children with amblyopia; those with multiple risk factors are counted repeatedly

### Grade 2 vision screening

A total of 2727 children were eligible for vision screening in Grade 2 (50 schools), and the parents of 47 children (1.7%) opted out. Overall, 1193 (43.7%) children had normal vision, 1327 (48.7%) were referred for follow-up eye exams, and 160 (5.9%) were absent. If universal screening can lead to successful treatment of amblyopia and other visual problems, we would expect to see a difference in prevalence rates between screened and non-screened schools. However, the prevalence of suspected amblyopia in screened schools (8.6%) did not differ statistically from the prevalence in non-screened schools (7.5%) (*t*(48) = 1.7, *p* = 0.10). In addition, the prevalence of “other” visual problems (e.g., reduced stereoacuity, refractive errors; see Table 3) did not differ statistically between screened schools (45.5%) and non-screened schools (47.1%) (*t*(48) =  − 0.67, *p* = 0.51). Thus, screening in SK did not appear to lower the prevalence of visual problems in Grade 2 (Table 5). Not surprisingly, there was no difference between screened and non-screened schools in reading nor math scores (*F*(47) = 0.03, *p* = 0.86) (see the Appendix Fig. 3). In screened schools, 33% of children were reading 1 SD below grade level, compared to 29% in non-screened schools.

**Table 5:** Comparison of the visual outcomes in Grade 2 at 25 screened and 25 non-screened schools

Variable of interest	Screened schools (% screened)	Non-screened schools (% screened)	*t*-value (*df* = 48)	*p*-value
Amblyopia	8.6%	7.5%	1.70	0.10
Problem other than amblyopia	45.1%	47.1%	− 0.67	0.51
Missing glasses	8.3%	4.7%	2.55	0.01
Wearing glasses	10.2%	7.8%	2.05	0.05

Where we did observe a difference between screened vs. non-screened schools was in the number of children wearing glasses (10.2% vs. 7.8%, *t*(48) = 20.5, *p* = 0.05), and the number of children who reported that their glasses were currently missing (e.g., at home, lost, or broken) (8.3% vs. 4.7%, *t*(48) = 2.55, *p* = 0.01). These results confirm that SK screening had identified more children with visual problems (and provided treatment in the form of glasses) than the status quo. Among the 169 children who reported their glasses missing in screened schools, 151 (89.3%) failed Grade 2 vision screening, suggesting that wearing their glasses could have been helpful.

Table 6 shows the number of children with visual problems in Grade 2 combined across screened and non-screened schools. The surprisingly high estimate of children with a visual problem (53.8%) appears to stem mainly from high rates of astigmatism and reduced stereoacuity.

**Table 6:** Prevalence of visual problems among screened Grade 2 children in entire sample

Disorder	Number of cases	(% screened)
Any problem	1356	53.8%
Amblyopia	199	7.9%
Bilateral amblyopia	62	2.5%
Unilateral amblyopia	137	5.4%
Reduced stereo (> 40 arcsec)	608	24.1%
Reduced acuity (20/32 or worse)	439	17.4%
Untreated refractive error	1007	40.0%
Hyperopia ≥ 1.25 D	270	10.7%
Myopia ≤ − 1.25 D	53	2.1%
Astigmatism ≥ 0.75 D	936	37.1%
Anisometropia	95	3.8%

The pattern of results did not differ when we accounted for attrition by re-calculating prevalence based only on Grade 2 children enrolled at the school when we began vision screening (83.3% of original sample). Of the children identified as having *any* visual problem in SK, only 51% of the sample were still at the same school and re-assessed in Grade 2. Simultaneously, 237 children who had not received SK screening had newly entered the “screened” schools, constituting 17% of the Grade 2 population, again reducing power when estimating benefits at the *school* level. With the sample restricted to those in school in both SK and Grade 2, there was still no difference between screened vs. non-screened schools in the prevalence of suspected amblyopia (8.5% vs. 7.3%, *t*(48) = 1.04, *p* = 0.32), nor visual problems other than amblyopia (44.2% vs. 47.6%, *t*(48) =  − 1.10, *p* = 0.28). There was only a trend for more children to be wearing glasses in screened schools (11.0% vs. 8.4%, *t*(48) = 1.84, *p* = 0.07), and again, more children reported their glasses missing in screened schools than in non-screened schools (8.6% vs. 5.0%, *t*(48) = 2.25, *p* = 0.03). Specifically, among the 82 children diagnosed with amblyopia in SK, 25 (30.5%) reported their glasses missing in Grade 2, indicating low compliance with treatment (9/25 children missing glasses still had suspected amblyopia in Grade 2).

## Discussion

The goal of the present study was to determine whether a school-based screening program comprised of screening, follow-up eye examinations, and provision of glasses to kindergarten children can reduce the later prevalence of visual problems. The program identified successfully over 300 senior kindergarten children with visual problems and provided glasses to them when needed. However, 1.5 years after screening, there was no difference between screened vs. non-screened schools, perhaps because 20% of screened children (and 51% of children with a visual problem) had moved away before Grade 2 screening. Additionally, our study revealed significant barriers to care, with almost 50% of children who had received glasses in SK reporting that, by Grade 2, their glasses were not worn regularly or had been lost or broken and never replaced. These findings are consistent with recent systematic reviews of vision screening that found that children were more likely to be wearing glasses at an unexpected follow-up visit if the glasses had been provided than if the parents had only received a letter with the prescription; however, this effect was reduced if the follow-up was 6‒12 months later (Wu et al., 2023).

Previous research has demonstrated that amblyopia can be treated effectively with early intervention and good compliance. Our results are consistent with such findings, as 59/82 children diagnosed with amblyopia in SK and tested on acuity in Grade 2 no longer had amblyopia. Thus, early intervention is effective at the *individual* level of analysis. To the extent that *individual children* can continue to access eye care easily, we believe there is a benefit to conducting school-based vision screening. However, barriers exist, as although every effort was made to facilitate follow-up care beyond the first eye exam (providing contact info, setting up appointments, sending reminders for follow-up appointments), we reached very few parents. In fact, we communicated with only 7 families out of the roughly 300 SK children who had a visual problem or concern. These results underscore the barriers to health care that children face, whether it be busy parents, lack of parents’ awareness about the importance of treatment compliance, poor accessibility of eye care, and/or parents’ concerns about payment for glasses, even when a research program is providing the glasses (including replacements) at no charge. Previous research reveals that children from lower-income neighbourhoods face more barriers in accessing health care (Johnson et al., 2000), and in the current study, 54% of Grade 2 children in high-needs schools had a visual problem, a rate much higher than our previous estimates of visual problems among kindergarten children (27% in high-needs schools in Toronto and 11% across Ontario; Nishimura et al., 2020). The high rate suggests that the status quo is insufficient to help children in need. Collectively, these findings reveal the need for a targeted approach to identify children with visual problems from low-income families and provide a comprehensive program of vision care, including rescreening in Grade 1, frequent follow-up support, tracking families as they move, and alerting teachers as to which children should be wearing glasses. In fact, providing a second pair of glasses that stays at school for when the child forgets them at home, or loses/breaks them, has been shown to be an effective intervention (Ethan et al., 2010).

Our hypothesis was that the treatment with glasses would benefit children whose reading had been hindered by their amblyopia or refractive errors (Pirindhavellie et al., 2023), such that fewer children would be reading below grade level in screened as compared to non-screened schools. However, many children who received glasses in SK had moved away or were no longer wearing them in Grade 2. Perhaps for that reason, we found no benefit of the intervention on reading skills. One previous study conducted in Florida compared reading outcomes among children in Grades 4‒5 in schools that did not receive screening, that received screening only, or that received screening + follow-up eye exams + glasses, and found better academic achievement among children in schools receiving screening + exams + glasses (Glewwe et al., 2018). Our results expand on this previous finding to suggest that continued access to care and glasses replacement are important in demonstrating long-term benefits at the population level.

### Limitations

One possible explanation for the lack of screening benefit is insufficient time for treatment. Some children received their glasses as they began Grade 1, meaning that they had worn their glasses for little more than a year; perhaps 2 years is necessary to demonstrate a benefit. However, these children could also move to another school or lose their glasses during that additional year, making it difficult to demonstrate any benefit at the school level.

Because eye examinations with cycloplegia were not performed in Grade 2, we may have missed some cases of hyperopia. However, the Spot autorefractor has a very high correlation with cyclopleged retinoscopy (for children aged 3–9 years), and especially good accuracy for identifying astigmatism (Peterseim et al., 2014), the most common type (37%) of visual problem identified in our sample. Thus, a possible underestimation of hyperopia likely had minimal impact on our results.

## Conclusion

With early intervention and good treatment compliance, amblyopia and refractive errors can be treated effectively. We tested whether a school-based vision screening would lead to a greater reduction in visual problems in schools that received the program than in schools that did not receive such intervention. We did not observe a difference between screened vs. non-screened schools, possibly because nearly half of the children diagnosed with amblyopia in SK had moved out of the sample by follow-up. As well, more children were missing their glasses in screened than in non-screened schools, suggesting that parents did not replace lost or broken glasses. Thus, the status quo (annual well-child visits, SK vision screening in school, and OHIP-covered eye exams) and additional program benefits (e.g., free glasses) are not enough to ensure adequate treatment for disadvantaged children. Nevertheless, 72% of children identified in SK as having amblyopia no longer had amblyopia in Grade 2, which indicates the need for effective early intervention and treatment. The success of a visual health intervention will depend on a multi-pronged approach to improve public awareness of early vision care through complementary communications from physicians, eye care professionals, public health, and educators involving vision screening at *all* or *targeted* schools, and the continued provision of follow-up care and glasses to *individual* children who need them.

## Contributions to knowledge

What does this study add to existing knowledge?

- School-based vision screening followed by an eye exam by an optometrist and a free pair of glasses is not enough to ensure adequate treatment for kindergarten children in high-needs schools.

What are the key implications for public health interventions, practice, or policy?

- For public health policy makers: school-based vision screening is an effective strategy to identify young children with visual problems, but the program must include follow-up treatments and continued access to affordable glasses to reduce the prevalence of vision problems in children.
- Ontario public health: provide glasses to children from low-income families.
- For eye care professionals and public health personnel involved in children’s care: continue to advocate for strategies that allow children, especially those from low-income families, to have glasses paid for them through their provincial health insurance plans.
- For parents: increase awareness about the importance of continuing eye care for their children.

## Figures and Tables

**Fig. 3:**
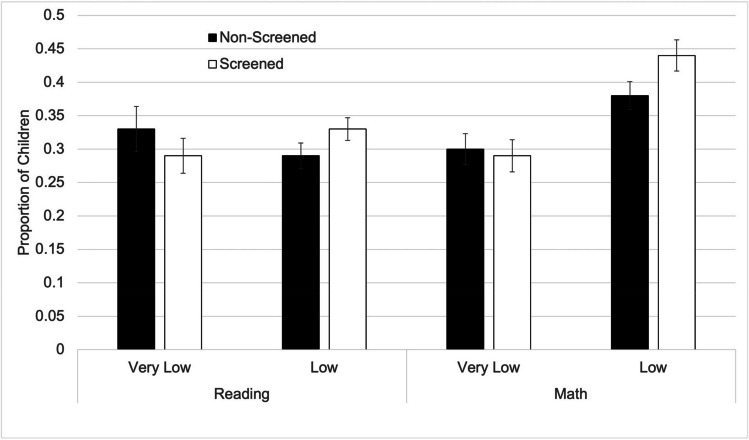
Proportion of children performing below grade average on reading and math assessments (very low = 2SD below average, low = 1SD below average) in non-screened and screened schools. These data include children in “screened” schools who had not been screened in SK (e.g., opted out, away during screening, not yet enrolled at that school), because data were provided to us aggregated *per school* by the school board

## Data Availability

The full data set is available on request from the corresponding author at nishimm@mcmaster.ca or senior author DM at maurer@mcmaster.ca until 7 years after publication, at which time the data will be destroyed and deleted in accordance with data management policies of SickKids Hospital. The trial is registered at clinicaltrials.gov (ID 1000045972).
